# Supplementary material for: Genome-wide association study identifies two risk loci for tuberculosis in Han Chinese
Source: Nat Commun. 2018 Oct 4;9:4072. doi: 10.1038/s41467-018-06539-w (PMC6172286; doi:10.1038/s41467-018-06539-w)
Supplement: Supplementary file 1 — Supplementary Information [file 41467_2018_6539_MOESM1_ESM.pdf]

Supplementary Information

Genome-wide association study identifies two new risk loci for TB in Han Chinese

Ruijuan Zheng[1#], Zhiqiang Li[2,3#], Fusheng He[4#], Haipeng Liu[1]*, Jianhua Chen[3], Jiayu Cheng[5], Xuefeng Xie[4], Juan Zhou[3], Hao Chen[6], Xiangyang Wu[6], Juehui Wu[6], Boyu Chen[3], Yahui Liu[3], Haiyan Cui[7], Lin Fan[7], Wei Sha[7], Yin Liu[8], Jiqiang Wang[3], Xiaochen Huang[1], Linfeng Zheng[5], Feifan Xu[9], Jie Wang[1], Yonghong Feng[1], Lianhua Qin[1], Hua Yang[1], Zhonghua Liu[1], Zhenglin Cui[1], Feng Liu[1], Xinchun Chen[10], Shaorong Gao[5], Silong Sun[4]*, Yongyong Shi[2,3,11]*&Baoxue Ge[1,6]*

# Equal contributions.
* Corresponding author

Supplementary Table 1. Characteristics of TB patients and controls.

	Group	Number	Age (Mean±SD)	% Female
GWAS	TB	833	45.32±7.74	45.14%
	Control	1,220	46.94±5.45	45.66%
REP I	TB	1,074	40.77±5.43	45.74%
	Control	1,904	45.05±15.64	47.01%
REP II	TB	1,042	42.69±3.62	37.66%
	Control	1,966	43.54±14.80	59.26%

Supplementary Table 2. Power analysis for the total sample size used in this study.

**Risk Allele Frequency**	**Genotype Relative Risk**						
	**1.10**	**1.20**	**1.30**	**1.40**	**1.50**	**1.75**	**2.50**
**0.010**	0.000	0.000	0.000	0.001	0.003	0.046	**0.863**
**0.030**	0.000	0.000	0.005	0.042	0.182	0.836	**1.000**
**0.050**	0.000	0.002	0.037	0.242	0.632	**0.997**	**1.000**
**0.100**	0.000	0.022	0.332	**0.853**	**0.993**	**1.000**	**1.000**
**0.200**	0.001	0.188	**0.880**	**0.999**	**1.000**	**1.000**	**1.000**
**0.350**	0.004	0.475	**0.990**	**1.000**	**1.000**	**1.000**	**1.000**
**0.500**	0.006	0.556	**0.995**	**1.000**	**1.000**	**1.000**	**1.000**
**0.750**	0.002	0.245	**0.911**	**0.999**	**1.000**	**1.000**	**1.000**
**0.900**	0.000	0.014	0.209	0.667	**0.941**	**1.000**	**1.000**
**0.970**	0.000	0.000	0.002	0.016	0.066	0.435	**0.995**

Power was estimated for the total sample size used in our study giving a range of Risk Allele Frequency and Genotype Relative Risk and assuming a population prevalence of 0.004 and a significance level of 5×10^{-8}. An adequate power of 85% or greater was indicated in bold.

Supplementary Table 3. Results for the 10 tested SNPs in the replication stages.

Chr.	SNP	Position (hg19)	Effect allele	Meta-analysis			Replication 1				Replication 2			
				OR	95% CI	P-value	Freq.	OR	95% CI	P-value	Freq.	OR	95% CI	P-value
2	rs7575462	80,012,056	T	0.994	0.918-1.076	0.8810	0.335	1.047	0.935-1.173	0.4231	0.345	0.9443	0.844-1.057	0.3175
2	rs62197322	232,061,520	G	0.921	0.852-0.996	0.0386	0.379	0.953	0.853-1.065	0.3969	0.398	0.8909	0.799-0.994	0.0386
3	rs11924733	72,106,014	A	0.945	0.873-1.023	0.1653	0.351	0.867	0.773-0.971	0.0135	0.351	1.0270	0.919-1.148	0.6409
5	rs1666780	8,533,701	T	1.082	1.003-1.167	0.0417	0.439	1.078	0.968-1.201	0.1706	0.436	1.0860	0.976-1.208	0.1297
6	rs3729618	45,888,073	G	0.911	0.816-1.018	0.1000	0.455	0.911	0.816-1.018	0.1001	-	-	-	-
11	rs10833753	22,432,279	T	0.957	0.852-1.075	0.4571	0.335	0.957	0.852-1.075	0.4575	-	-	-	-
12	rs715948	57,532,982	C	1.071	0.993-1.156	0.0756	0.429	1.088	0.977-1.212	0.1265	0.457	1.0550	0.948-1.174	0.3263
12	rs11176643	67,629,786	G	0.992	0.917-1.074	0.8437	0.341	1.017	0.908-1.139	0.7638	0.358	0.9685	0.867-1.082	0.5725
14	rs12437118	76,983,730	A	1.260	1.160-1.369	4.65E-08	0.269	1.292	1.150-1.452	1.72E-05	0.261	1.2280	1.092-1.381	5.91E-04
20	rs6114027	2,379,598	C	1.296	1.174-1.430	2.62E-07	0.160	1.285	1.116-1.480	4.92E-04	0.158	1.3060	1.138-1.499	1.45E-04

Genotyping of rs3729618 and rs10833753 failed in the replications 2 stage because of poor clusters.

Supplementary Table 4. Results for the previously identified TB associated SNPs.

Locus	Chr.	SNP	Position (hg19)	Effect allele	Freq.	Discovery stage		Previous reports		
						OR (SE)	P	OR [95% CI]	P	Freq.
ASAP1	8	rs10956514	131,252,758	G	0.637	0.948 (0.069)	0.442	0.85 [0.81-0.89]	1.00E-10	0.358
ASAP1	8	rs4733781	131,296,767	C	0.639	0.940 (0.069)	0.369	0.84 [0.80-0.88]	2.60E-11	0.314
11p13	11	rs2057178	32,364,187	A	0.046	0.882 (0.167)	0.453	0.82 [0.77-0.86]	2.57E-11	0.110-0.320
18q11.2	18	rs4331426	20,190,795	G	0.024	1.192 (0.210)	0.403	1.19 [1.12-1.26]	6.80E-09	0.429-0.525
HLA	6	rs557011	32587013	T	0.483	1.192 (0.068)	9.79E-03	1.25 [1.17-1.33]	5.80E-12	0.402
HLA	6	rs9271378	32587300	G	0.280	0.856 (0.077)	0.043	0.78 [0.73-0.84]	2.50E-12	0.325
HLA	6	rs9272785	32610401	A	0.304	0.981 (0.073)	0.795	1.22 [1.13-1.32]	3.50E-07	0.191

Supplementary Table 5. Allele frequencies for rs12437118 and rs6114027 in different populations

Chr.	SNP	Position	A1	A2	AFR Freq.	AMR Freq.	ASN Freq.	EUR Freq.
14	rs12437118	76983730	A	G	0.30	0.32	0.30	0.35
20	rs6114027	2379598	C	T	0.23	0.16	0.17	0.10

The allele frequencies were obtained from the 1000 Genomes Phase 1 data for African (AFR), American (AMR), Asian (ASN), and European (EUR) populations.

Supplementary Table 6. Characteristics of TB patients.

Characteristic	Genotype			P value
	TT	TC	CC	
	(n=704)	(n=377)	(n=73)	*P* value
Age (Mean±SD)	42.43±18.81	42.89±17.59	40.22±15.94	0.4382[*]
Male, number (%)	488(69)	256(67)	51(69)	0.8766[#]

[*] $\chi 2$ test, data are displayed as percent of individuals.

[#] one-way ANOVA for statistical analyses.

Supplementary Table 7. Nucleotide sequences for primers used in functional assessments.

**Primer**	**Sequence (5' to 3')**
Tgm6 sgRNA-1	TGCTGTCTGGAATACGGCTT
Tgm6 sgRNA-2	GTATCGGCCAATGACTGCAT
m-*tgm6* forward for genotyping	GGCCCACAGACTACCGATTT
m-*tgm6* reverse for genotyping	GTCTGACTCTGGGGACCTCT
m-*tgm6* forward-1	ACACCCAAGATTACCCTTGCT
m-*tgm6* reverse-1	GGATGCGTGAGGTCCTGTC
m-*tgm6* forward-2	AAGCCGTATTCCAGACAGCA
m-*tgm6* reverse-2	CATTGGAAGGGCTCGAGAGG
m-*il-10* forward	GCTCTTACTGACTGGCATGAG
m-*il-10* reverse	CGCAGCTCTAGGAGCATGTG
m-*il-1β* forward	GCAACTGTTCCTGAACTCAACT
m-*il-1β* reverse	ATCTTTTGGGGTCCGTCAACT
m-*il-6* forward	TCCAGTTGCCTTCTTGGGAC
m-*il-6* reverse	GTGTAATTAAGCCTCCGACTTG
m-*il-12* forward	GAGCACTCCCCATTCCTACT
m-*il-12* reverse	CCCTCCTCTGTCTCCTTCAT
m-*inf-γ* forward	ACAGCAAGGCGAAAAAGGATG
m-*inf-γ* reverse	TGGTGGACCACTCGGATGA
m-*tnf-α* forward	CTGAACTTCGGGGTGATCGG
m-*tnf-α* reverse	GGCTTGTCACTCGAATTTTGAGA
m-*ccl-2* forward	TTAAAAACCTGGATCGGAACCAA
m-*ccl-2* reverse	GCATTAGCTTCAGATTTACGGGT
m-*ccl-3* forward	TGTACCATGACACTCTGCAAC
m-*ccl-3* reverse	CAACGATGAATTGGCGTGGAA
m-*cxcl-11* forward	TGTAATTTACCCGAGTAACGGC
m-*cxcl-11* reverse	CACCTTTGTCGTTTATGAGCCTT
h- *ESRRB* forward	TCGCTGCCCTATGACGACA
h- *ESRRB* reverse	CTTCTTGTACCTGCGTACCAG
h- *TGM6* forward	TCAGGCTTTCCTCTCACCG
h- *TGM6* reverse	CTGAGCACGTACTCCTGTCTC

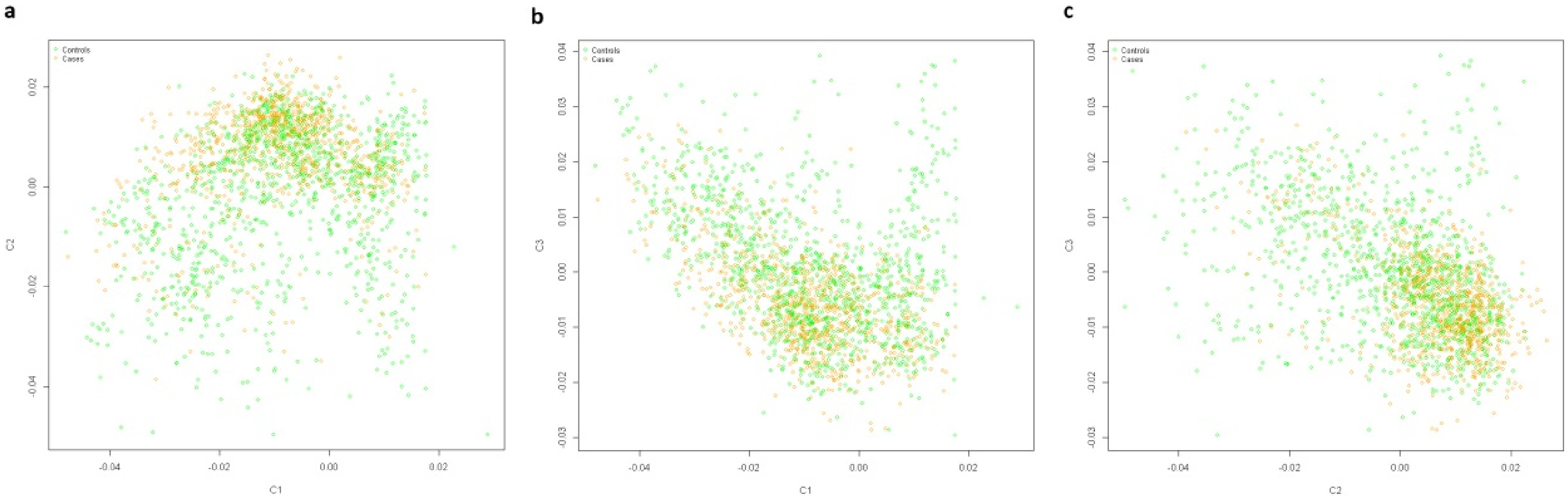

Supplementary Figure 1. PCA analysis of the GWAS sample. (**a**) The first principal component (C1, x axis) was plotted against the second principal component (C2, y axis). (**b**) The first principal component (C1, x axis) was plotted against the third principal component (C3, y axis). (**c**) The second principal component (C2, x axis) was plotted against the third principal component (C3, y axis). Controls are green circles and Cases are yellow circles.

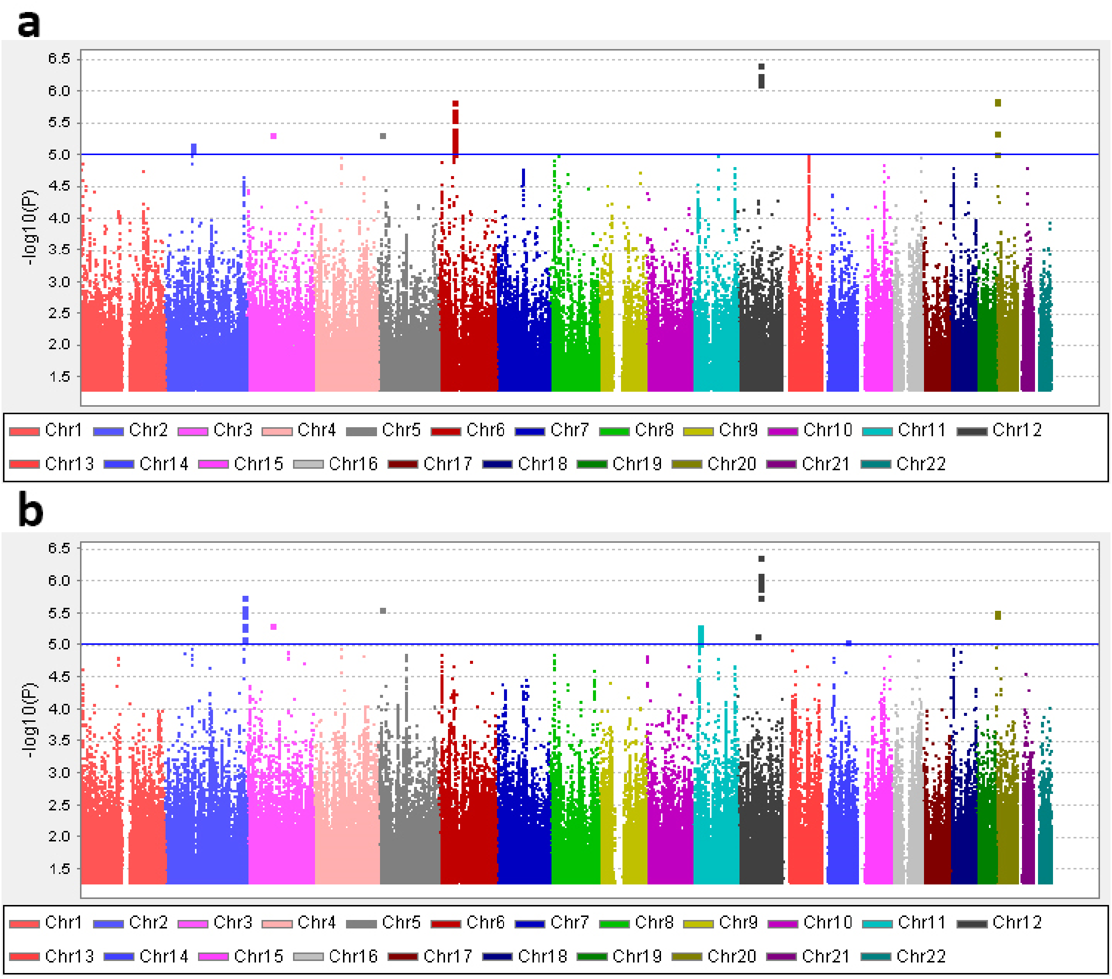

Supplementary Figure 2. Manhattan plot of the GWAS analysis. **(a)** PCA-based analysis; **(b)** unadjusted analysis. Genome-wide P-values ($-\log_{10}P$, y axis) plotted against their respective chromosomal positions (x axis). The blue line is the significance level used for follow up (1×10^{-5}). SNPs with P-values greater than 0.05 were not shown.

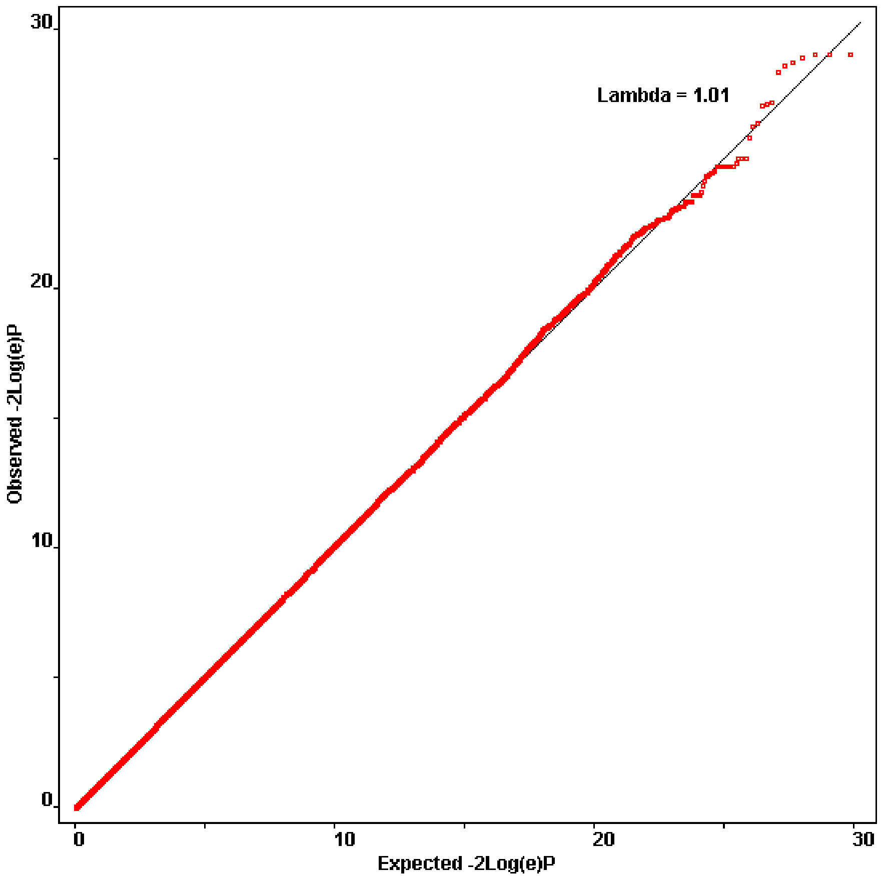

Supplementary Figure 3. Q-Q plot of the GWAS analysis. The quantile-quantile plot representative of observed (y axis) vs. expected (x axis) SNPs distribution. The plot was generated on PCA adjusted data.

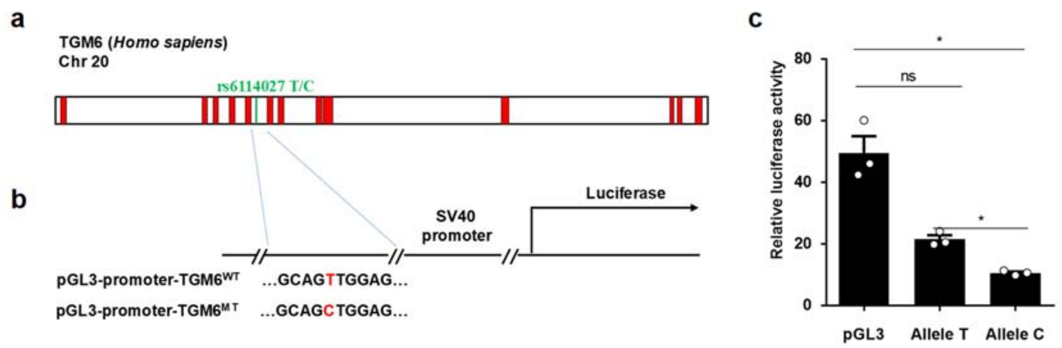

Supplementary Figure 4. Effect of SNP rs6114027 on promoter activity. (**a**) Schematic presentation of the location of SNP rs6114027. (**b)** Schematic drawing of SNP rs6114027 promoter luciferase reporter constructs (pGL3-promoter-*Tgm6*). The intron between exon 5 and 6 containing 1514 bp with SNP rs6114027 T or C allele was cloned into luciferase reporter vector (pGL3-promoter). (**c**) Luciferase reporter plasmids containing either rs6114027 T or C allele or the control plasmid were transfected into HEK293T cells and relative luciferase activities were assayed by using *Renilla* luciferase activity for normalization. Data shown are mean ± SEM of n=3 independent experiments. One-way ANOVA followed by the Bonferroni post hoc test were used for statistical analyses. *, $P<0.05$; ns, not significant.

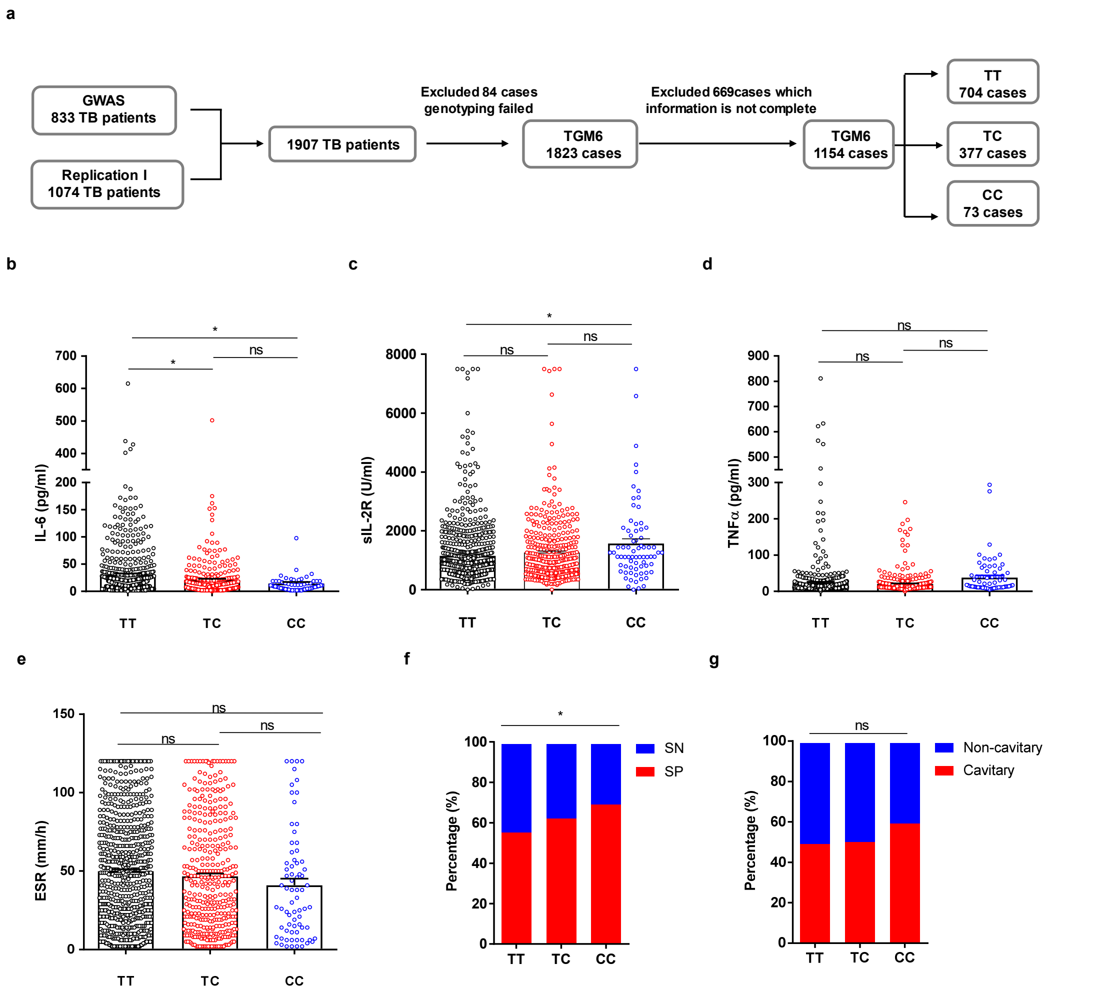

Supplementary Figure 5. Association of *TGM6* SNP with clinical indices in TB patients. (**a**) Schematic flow chart of the study and filtration strategy for the analysis between SNP and clinical indices. (**b-e)** The association between rs6114027 genotype with the abundance of IL-6 (**b**), sIL-2R(**c**), TNF-α (**d**) and ESR (**e**) in the plasma. Data shown are mean ± SD of indicated number of individuals (TT, n=704; TC, n=377; CC, n=73.). One-way ANOVA followed by the Bonferroni post hoc test were used for statistical analyses. (**f**) The association between rs6114027 genotype with the presence the *Mtb* in the sputum smear as detected by acid-fast staining. (**g**) The association between rs6114027 genotype with the presence of cavity in the lung. (TT, n=704; TC, n=377; CC, n=73.). The χ^2 test were used to compare variables shown as percentages (**f, g**). *, P<0.05; ns, not significant. ESR, erythrocyte sedimentation rate; SN, sputum smear negative; SP, sputum smear positive.

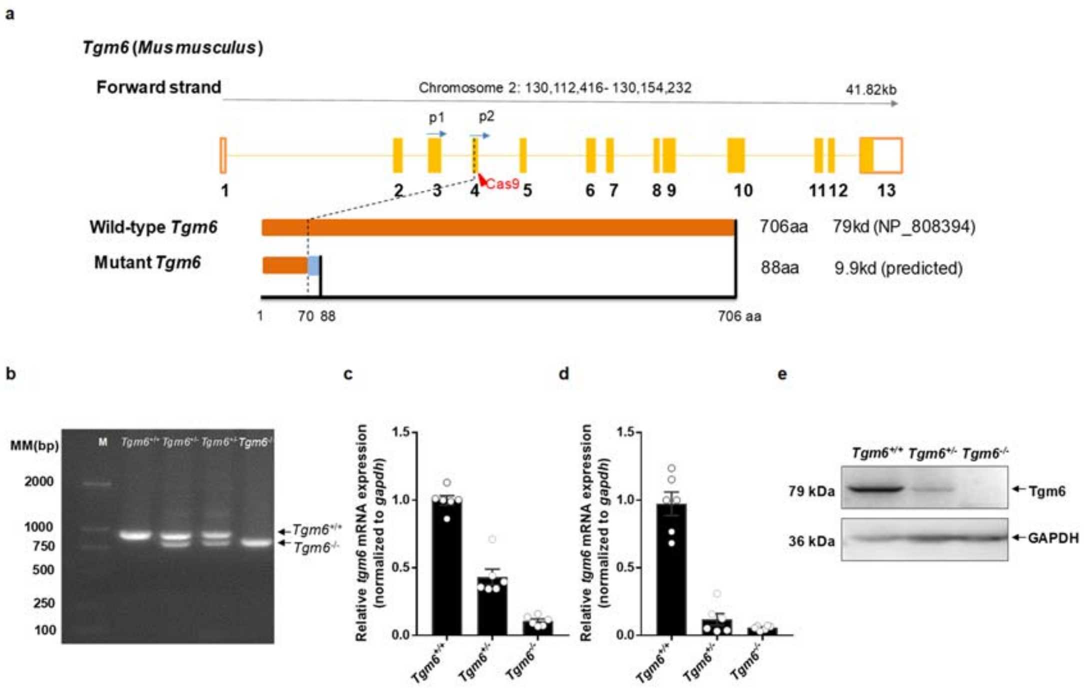

Supplementary Figure 6. Generation of *tgm6* knockout mice by CRISPR/Cas9-mediated genome editing. (**a**) Schematic description of *tgm6* gene structure and strategy to generate *tgm6* knockout mice by CRISPR/Cas9-medited genome editing. Red arrowhead indicates the targeting sites of the sgRNA. Wild type *tgm6* possesses 706 amino acids (aa); the deletion of 110 nucleotides located in the exon 4 of *tgm6* caused a reading frame shift and a premature stop codon. (**b**) Genotyping of wild type and *tgm6* mutant mice as detected by PCR. MM, molecular marker. (**c-d**) qPCR detection of *tgm6* transcripts in the lungs from mice of all 3 genotypes with two different probes. The probe 1 was designed to target exon 3 of *tgm6* (**c**) and the probe2 was designed to target the deleted sequence located in the exon 4 of *tgm6* (**d**). Data shown were mean ± SEM of n=6 mice. (**e**) Western blot detection of tgm6 protein in the lysates of lungs from mice of all 3 genotypes.

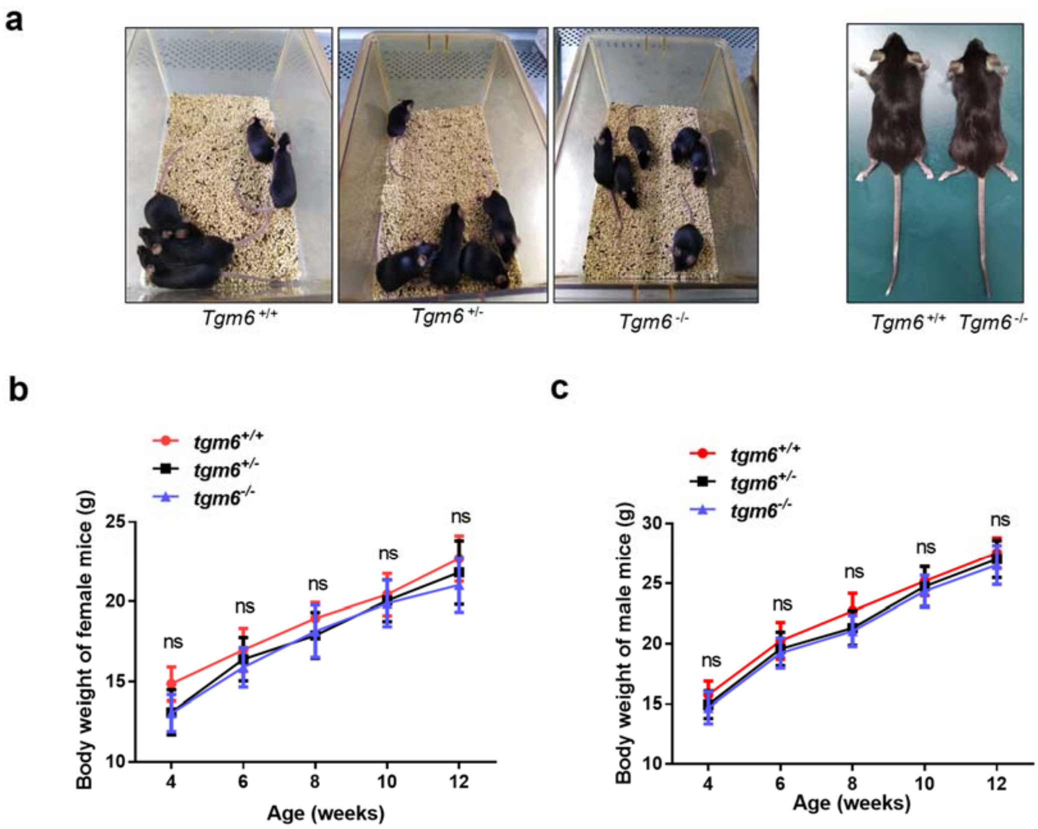

Supplementary Figure 7. Gross phenotype of *tgm6* mutant mice. (**a**) Gross phenotype of 7-8 weeks old female mice of 3 different genotypes. (**b-c**) The curve of body weight of male (**b**) and female (**c**) mice of all 3 *tgm6* genotypes. Data shown are mean ± SD of n=4 mice and are representative of n=3 independent experiments. Two-way ANOVA followed by the Bonferroni post hoc test were used for statistical analyses. ns, not significant.

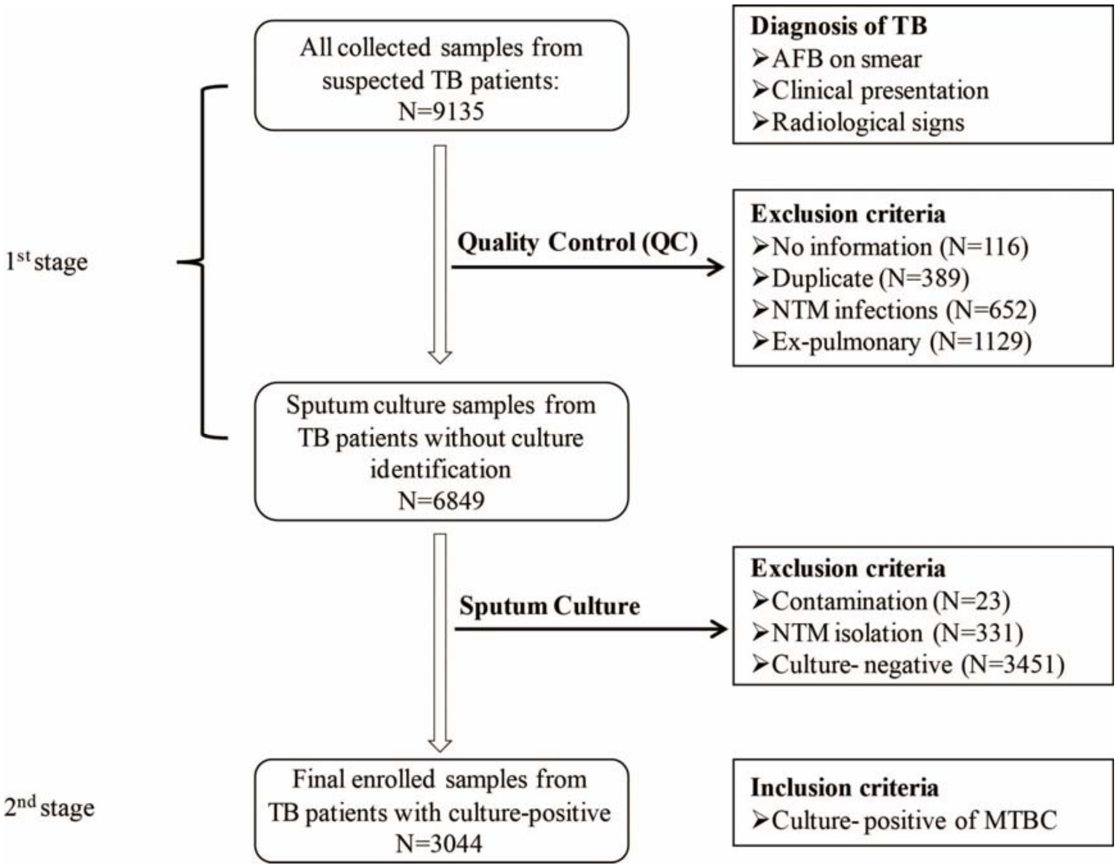

Supplementary Figure 8. Flow chart of the sample collection of TB patients. AFB, acid-fast bacilli; NTM, nontuberculous mycobacteria. MTBC, *M. tuberculosis* complex.

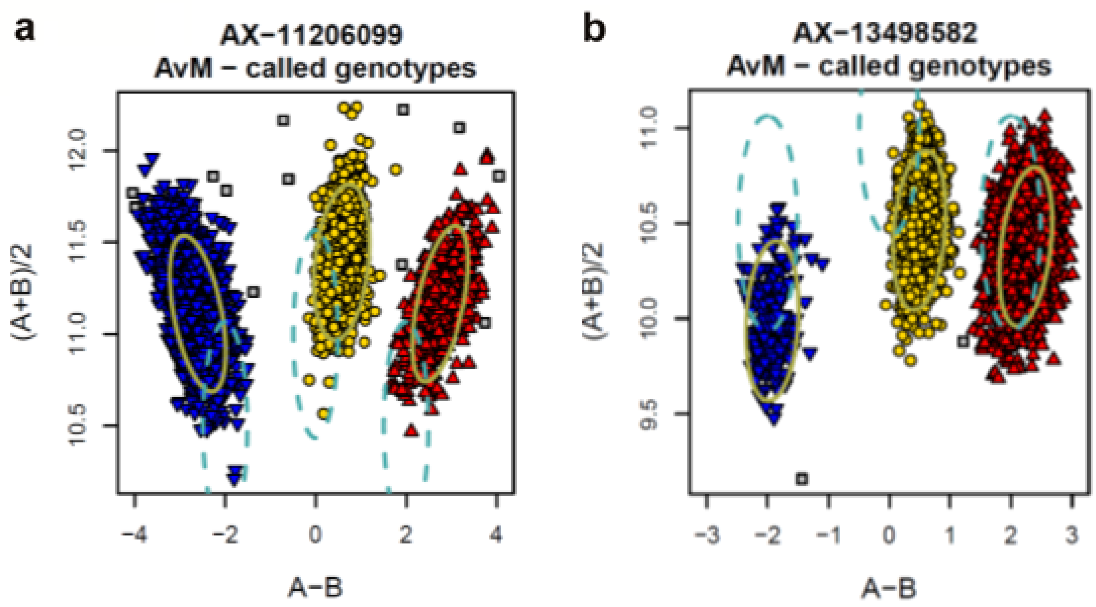

Supplementary Figure 9. Genotype cluster plots of the genome-wide significant SNPs for the discovery stage. (**a**) rs12437118 (Affymetrix Probe Set ID: AX-11206099). (**b**) rs6114027 (Affymetrix Probe Set ID: AX-13498582). The cluster plots, generated by the Ps_Visualization of Affymetrix SNPolisher package, showed the scatter pattern of transformed A and B channel intensity contrasts vs. the average of the transformed A and B channel intensities (Contrast vs Size). The colors for AA, AB and BB are red, gold and blue, respectively. No Calls are in grey.

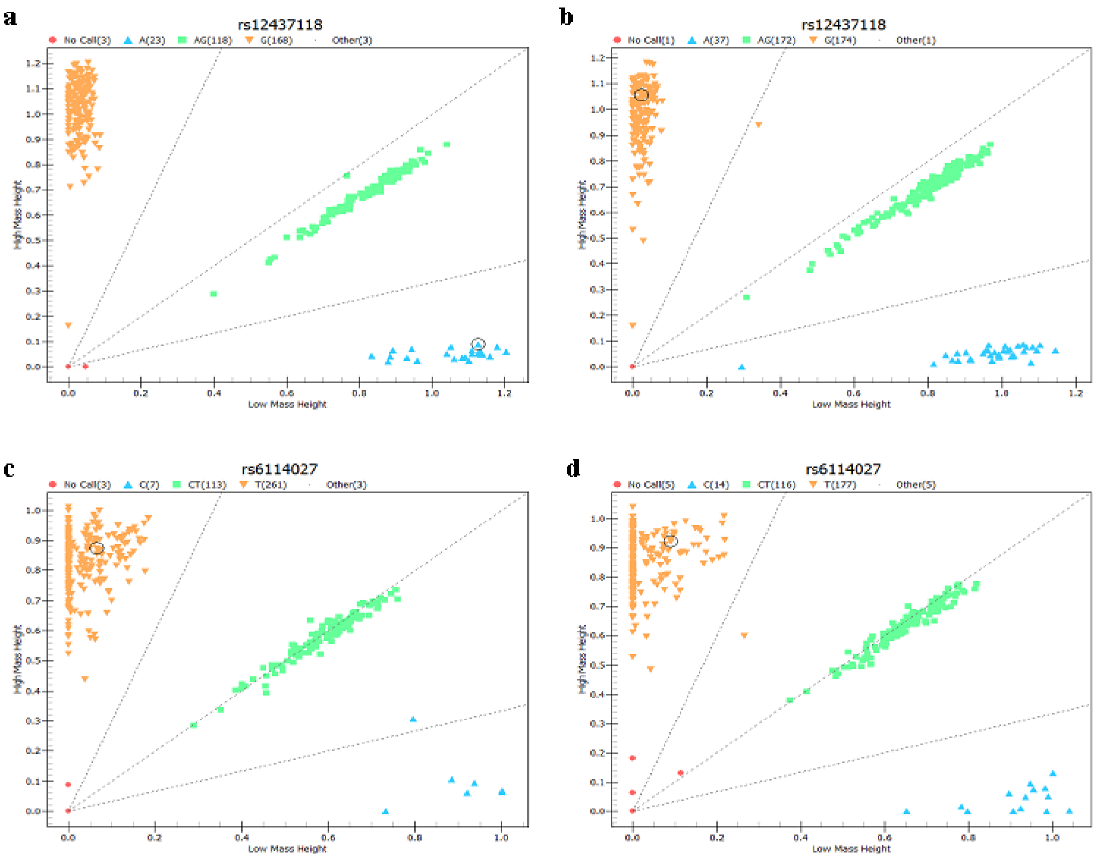

Supplementary Figure 10. Genotype cluster plots of the genome-wide significant SNPs for the replication stages. (**a**) rs12437118 (Replication 1). (**b**) rs6114027 (Replication 1). (**c**) rs12437118 (Replication 2). (**d**) rs6114027 (Replication 2). The Call Cluster plot, generated by MassARRAY Typer v4.0, showed the clustering of the individual genotypes based on the calls for each marker in each plate. Different symbols and colors are used to indicate different calls. No Calls are in red.
