# Supplementary material for: Genome-wide association study identifies two risk loci for tuberculosis in Han Chinese
Source: Nat Commun. 2018 Oct 4;9:4072. doi: 10.1038/s41467-018-06539-w (PMC6172286; doi:10.1038/s41467-018-06539-w)
Supplement: Supplementary file 3 — Description of Additional Supplementary Files [file 41467_2018_6539_MOESM3_ESM.pdf]

Description of Additional Supplementary Files

File Name: Supplementary Data 1
Description: SNPs with P<1 × 10–5 in the discovery stage.

File Name: Supplementary Data 2
Description: Results for the epigenomic analysis.

File Name: Supplementary Data 3
Description: Association signals of imputed classical HLA alleles with TB in Chinese.

File Name: Supplementary Data 4
Description: Top significant genes in the gene-based analysis.

File Name: Supplementary Data 5
Description: Top significant gene sets in the pathway-based analysis.

File Name: Supplementary Data 6
Description: Nucleotide sequences for primers used in the Sequenom iPLEX SNP Genotyping.
